# Biased Signaling of the Angiotensin II Type 1 Receptor Can Be Mediated through Distinct Mechanisms

**DOI:** 10.1371/journal.pone.0014135

**Published:** 2010-11-30

**Authors:** Marie Mi Bonde, Jonas Tind Hansen, Samra Joke Sanni, Stig Haunsø, Steen Gammeltoft, Christina Lyngsø, Jakob Lerche Hansen

**Affiliations:** 1 Laboratory for Molecular Cardiology, The Danish National Research Foundation Centre for Cardiac Arrhythmia, The Heart Centre, Copenhagen University Hospital, Rigshospitalet, Copenhagen, Denmark; 2 Department of Biomedical Sciences and The Danish National Research Foundation Centre for Cardiac Arrhythmia, Faculty of Health Sciences, University of Copenhagen, Copenhagen, Denmark; 3 Department of Clinical Biochemistry, Glostrup Hospital, Glostrup, Denmark; University of Oldenburg, Germany

## Abstract

**Background:**

Seven transmembrane receptors (7TMRs) can adopt different active conformations facilitating a selective activation of either G protein or β-arrestin-dependent signaling pathways. This represents an opportunity for development of novel therapeutics targeting selective biological effects of a given receptor. Several studies on pathway separation have been performed, many of these on the Angiotensin II type 1 receptor (AT1R). It has been shown that certain ligands or mutations facilitate internalization and/or recruitment of β-arrestins without activation of G proteins. However, the underlying molecular mechanisms remain largely unresolved. For instance, it is unclear whether such selective G protein-uncoupling is caused by a lack of ability to interact with G proteins or rather by an increased ability of the receptor to recruit β-arrestins. Since uncoupling of G proteins by increased ability to recruit β-arrestins could lead to different cellular or *in vivo* outcomes than lack of ability to interact with G proteins, it is essential to distinguish between these two mechanisms.

**Methodology/Principal Findings:**

We studied five AT1R mutants previously published to display pathway separation: D74N, DRY/AAY, Y292F, N298A, and Y302F (Ballesteros-Weinstein numbering: 2.50, 3.49–3.51, 7.43, 7.49, and 7.53). We find that D74N, DRY/AAY, and N298A mutants are more prone to β-arrestin recruitment than WT. In contrast, receptor mutants Y292F and Y302F showed impaired ability to recruit β-arrestin in response to Sar^1^-Ile^4^-Ile^8^ (SII) Ang II, a ligand solely activating the β-arrestin pathway.

**Conclusions/Significance:**

Our analysis reveals that the underlying conformations induced by these AT1R mutants most likely represent principally different mechanisms of uncoupling the G protein, which for some mutants may be due to their increased ability to recruit β-arrestin2. Hereby, these findings have important implications for drug discovery and 7TMR biology and illustrate the necessity of uncovering the exact molecular determinants for G protein-coupling and β-arrestin recruitment, respectively.

## Introduction

**Figure 1:**
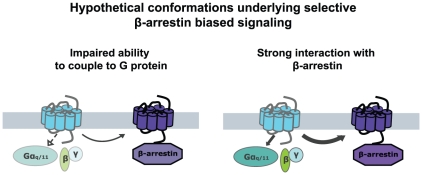
Schematic presentation of possible mechanisms underlying differential activation. Hypothetically, a receptor mutant selectively activating β-arrestin induced pathways could either be impaired in G protein-coupling (*right*) or show very strong interaction with β-arrestins (*left*).

Seven transmembrane receptors (7TMR) are surface receptors originally anticipated to signal only via heterotrimeric G proteins to second messengers such as inositol trisphosphates and cyclic AMP. It is now generally recognized that 7TMR signaling is much more diverse and that receptors can activate signaling pathways selectively mediated by multiple signaling conformations [1], [2]. This phenomenon is believed to have physiological relevance, hereby having widespread implications for both biological understanding and drug development. However, the molecular determinants underlying specific G protein or β-arrestin interactions have yet to be determined. Hypothetically, impaired G protein-dependent signaling and retained ability to interact with β-arrestins could be mediated by two different types of conformations: 1) One that lacks specific epitopes necessary for activating G proteins, but retaining those for other pathways, or 2) one that shows a preference for a G protein-independent pathway such as β-arrestin, which then prevents G protein-dependent signaling from occurring as illustrated in figure 1.

**Figure 2:**
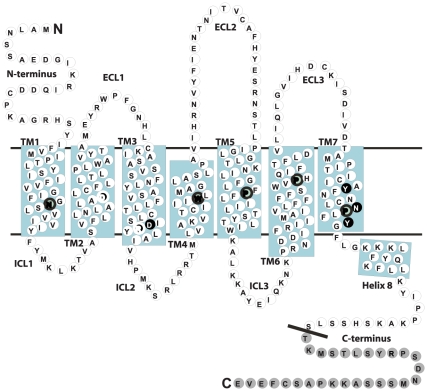
Schematic presentation of the residues targeted for mutations in a snake diagram of the rAT1aR. Residues mutated in this study are highlighted in black. These include the conserved DRY motif in the cytoplasmic part of TM3, the NPXXY motif in the cytoplasmic part of TM7, and the aspartic acid in TM2. Residues removed by truncation are shown in grey. The conserved residues indexed to the number 50 in the Ballesteros-Weinstein numbering scheme are highlighted in bold [5].

Activation of Family A 7TMRs most likely occurs through concerted movements of the helical bundle, which ultimately expose epitopes for intracellular signaling partners at the cytoplasmic surface [3], [4]. These movements are suggested to be facilitated by conformational changes of amino acids in the transmembrane domain that relieve structural constraints maintaining the inactive state. Several of these residues are conserved among Family A 7TMRs and include (numbered by the Ballesteros-Weinstein method [5]) an aspartic acid in TM2 (2.50), the DRY motif at the cytoplasmic part of TM3 (3.49–3.51), and the NPXXY motif in TM7 (7.49–7.53) [3], [4], see figure 2.

Functional selectivity proposes that receptors can adopt multiple conformations upon ligand binding [6]. This possibility allows different ligands and receptor mutants to selectively activate downstream signaling pathways [7]. Several of the studies of such functional selectivity or differential signaling have been conducted on the Angiotensin (Ang) II type 1 receptor (AT1R). An example of differential signaling is the recruitment of β-arrestins and the successive activation of β-arrestin-dependent ERK1/2 activation. This happens independently of G proteins and leads to different temporal and spatial distributions of the activated kinases, compared to the G protein-dependent response [7], [8], [9], [10], [11], [12].

G protein-independent signaling provides exciting opportunities in drug discovery. The AT1R is an important drug target due to its role in the regulation of salt and water homeostasis and cardiovascular functions [13], [14]. This is illustrated by the fact that AT1R antagonists or compounds inhibiting Ang II generation are efficient in the treatment of hypertension, cardiac hypertrophy, arrhythmias, and failure [14], [15]. Studies suggest that selective inhibition of Gα_q/11_ protein-dependent AT1R signaling may hold additional benefits for certain patients groups compared to the current un-biased treatment options [1]. Thus, determining the molecular events underlying differential signaling is essential for the design of selectively activating compounds. Several AT1R residues reported to differentially affect signaling pathways upon mutation are conserved polar residues in the transmembrane domain suggested to be involved in the conformational changes associated with receptor activation [12], [16], [17], [18], [19], [20].

As mentioned above and illustrated in figure 1, G protein-independent, β-arrestin-dependent signaling can hypothetically occur through either lack of ability to recruit G proteins or a very strong preference for β-arrestins. It has been shown that AT1R desensitization and internalization appear very robust compared to G protein activation [21]. Therefore, we speculated that at least some differentially activated AT1R mutants might be particularly prone to recruitment of β-arrestins. The AT1R interacts stably with β-arrestin and co-translocates with the molecule into cytosolic vesicles – a so-called Class B 7TMR pattern [22], [23]. Truncation or mutation of serine or threonine residues in the C-terminal tail has been shown to disrupt this stable interaction with β-arrestin leading to increased signaling through G protein-dependent pathways [24], [25], [26], [27], [28], [29].

In this study, we wanted to further investigate the conformational backgrounds of five different AT1R mutants, previously shown to be differentially activated in terms of loss of G protein-dependent signaling, but retaining ability to recruit β-arrestins, activate ERK1/2 and/or internalize – D74N, DRY/AAY, Y292F, N298A, and Y302F (Ballesteros nomenclature: 2.50, 3.49–3.51, 7.43, 7.49, 7.53, see figure 2) [12], [16], [17], [18], [19], [20], [30], [31]. To evaluate the effect of the stable interaction with β-arrestins, we truncated WT and mutant receptors after serine 331 (331Δ). Full length and truncated WT and mutant receptors were characterized in terms of inositol phosphate (IP) accumulation and β-arrestin2 recruitment. Our analysis revealed that receptor mutants D74N, DRY/AAY, and N298A showed more potent β-arrestin2 recruitment independently of G protein activation compared to WT receptor. This suggests that these receptor mutants are more prone to β-arrestin recruitment than WT. In contrast, receptor mutants Y292F and Y302F showed impaired ability to recruit β-arrestin in response to Sar^1^-Ile^4^-Ile^8^ (SII) Ang II suggesting that these receptor mutants represent conformational states distinct from both WT and the other receptor mutants tested in this study. Truncation improved ability to signal through Gα_q/11_ for all of the receptor mutants tested. However, the DRY/AAY mutant remained severely impaired in activation of G protein-dependent signaling. Together, these results provide new insights to the molecular determinants of differential signaling of the AT1R and further support the existence of multiple signaling states.

## Materials and Methods

### Ligands

Ang II was from Sigma Aldrich. Sar^1^-Ile^4^-Ile^8^ (SII) Ang II was synthesized at the Cleveland Clinic, Lerner Research Institute, OH, USA.

### Recombinant DNA plasmids

Mutations, except DRY/AAY, were generated by PCR with the WT rat AT1a as template using the QuickChange mutagenesis protocol (Stratagene) or as described by Heckman et al., 2007 [32]. Truncation was made by PCR either in parallel with mutation or separately on mutated background for constructs generated by Quickchange mutagenesis (Primer for truncation: 5′CGCTCTAGACTCGAGCTAAGACAGGCTTGAGTGGGACTTGGC3′). DRY/AAY was kindly provided by Dr. Laszlo Hunyady [17]. Constructs were subcloned or inserted into a modified pCDNA3.1 vector containing a FLAG-tag inserted after a hemagglutinin signal peptide [33]. For Renilla Luciferase (Luc)-tagged constructs, full length receptors were subcloned into a vector containing the Luc-tag [34]. For truncated constructs, stop codon was removed by PCR before insertion into the Luc-tagged vector (Primer: 5′CCTCTAGACTGGATCCAGAGACAGGCTTGAGTG3′). Mutations were verified by sequencing at Eurofins MWG Operon.

### Cell culture and transfection

COS-7 or HEK293 cells were grown in Dulbecco's modified Eagle's medium (DMEM) supplemented with 0.1 mg/mL Gentamicin (Invitrogen), 0.03% (w/v) L-Glutamine (Substrate Department at Panum Institute, Copenhagen, Denmark) and 10% (v/v) fetal bovine serum (BioChrome AG) in a humidified atmosphere at 37°C and 5% CO_2_. For transfection, cells were seeded in 10 cm dishes to obtain 80–90% confluence on the following day, where cells were transfected. For cell surface expression, competition binding, and inositol phosphate (IP) accumulation assays, COS-7 cells were transfected with 5 µg plasmid DNA using Lipofectamine2000 (Invitrogen). For β-arrestin2-recruitment, HEK293 cells were transfected with 1 µg of Luc-tagged AT1R and 3 µg of GFP^2^-β-arrestin2 using Polyethylenimine (PEI). For β-arrestin2-recruitment with overexpressed Gαq, HEK293 cells were transfected with 1 µg of Luc-tagged AT1R and 3 µg of YFP-β-arrestin2 in combination with 12 µg Gαq.

### Cell surface expression determined by whole cell ELISA

Transfected cells were seeded for triplicate measures with and without primary antibody in poly-lysine (Sigma-Aldrich) coated 96-well dishes at 30,000/well in 100 µL growth medium. Assay was conducted on the following day as described in Bonde et al., 2010 with minor modifications [35]. In brief, *for detection of surface expressed receptors*, cells were incubated with primary antibody (M1 anti-FLAG (Sigma-Aldrich), diluted 1∶1000) for 1h at 4°C. Cells were then fixed, blocked in 1% BSA and incubated with secondary antibody (HRP-conjugated anti-mouse IgG (GE Healthcare), diluted 1∶3000). *For detection of total protein*, blocking, primary, and secondary antibody conditions were conducted in the presence of 0.1% Triton X-100 for permeabilization. Assays were developed using 3,3′,5,5′-tetramethylbenzidine (TMB) liquid substrate system for ELISA (Sigma-Aldrich). Reactions were stopped with 1M hydrochloric acid after development of a blue color and absorbance at 450 nm was measured. Results were analyzed in GraphPad Prism and Excel. Triplicate means were normalized firstly by subtraction of values for cells without antibody, and secondly, by subtraction of values for mock transfected cells.

### Whole-cell competitive radio-ligand binding assay

Transfected cells were seeded in poly-lysine coated 48-well dishes at 100,000 cells/well for triplicate measures. On the following day, binding assay was conducted as described in Hansen et al., 2004 [36] with minor modifications. In brief, cells were incubated at 4°C for 30–60 mins, placed on ice and washed once in cold Hanks Balanced Salt Solution (HBSS) with 20 mM Hepes supplemented with 0.9 mM CaCl_2_ and 1.05 mM MgCl_2_ (HBSS^+^). Cells were incubated for 3h at 4°C with 1nM of ^3^H-Ang II (GE Healthcare) and increasing amounts of unlabeled Ang II diluted in HBSS^+^. Cells were washed twice in ice cold HBSS^+^ and lysed in lysis buffer (1% TritonX, 50 mM Tris-HCl pH 7.5, 100 mM NaCl, and 5mM EDTA) at room temperature (RT) for 30–60 mins of shaking. Well content was transferred to scintillation vials (Perkin Elmer) containing 4mL Ultima Gold scintillation liquid (Perkin Elmer). Total radioactivity was measured in a TriCarb 2800 TR Liquid Scintillation analyzer (Perkin Elmer). Results were analyzed in GraphPad Prism and Excel. Curves were fitted using the non-linear regression analysis (one-site competition) in GraphPad Prism. K_D_ values for Ang II were calculated using the equation K_D_ = IC_50_-[radioligand]. Statistical analysis, one-way ANOVA with Dunnett's post-test against full length WT was performed in GraphPad Prism. *For SII Ang II competition binding*, pK_i_ values were calculated based on K_D_ values for Ang II competition experiments conducted in parallel. DRY/AAY and N298A mutants (full and truncated) have been tested in a series of experiments separate from D74N mutants. Statistics were carried out separately for each set of experiments. Both datasets were analyzed by repeated measures ANOVA with subsequent Dunnet's multiple comparisons test against WT pK_i_ in GraphPad Prism.

### IP accumulation

Transfected cells were seeded in poly-lysine coated 96-well dishes at 50,000/well in 100 µL DMEM without I-inositol with Glutamax (Invitrogen) supplemented with 0.1 mg/mL Gentamicin (Invitrogen), 10% fetal bovine serum and 2 µCi H^3^myo inositol (Perkin Elmer) pr. mL medium. On the following day, IP assay was conducted as described in Bonde et al., 2010 [35]. Data were analyzed in GraphPad Prism and Excel. Statistical analysis was performed on pEC50 values and fitted maximum values in GraphPad Prism as described for competition binding above. DRY/AAY mutants were not included in this analysis. For DRY/AAY full length and 331Δ receptors were instead compared using Student's t-test (paired, two-tailed) in Excel on maximum response, fold over minimum response and amplitude (max.-min. response). Maximum response was calculated as an average of 0.1 and 1 µM responses, minimum response was the no drug condition. For the IP accumulation experiments of receptor mutants with or without Gαq overexpression, pEC50 values for WT, WT plus Gαq, D74N plus Gαq and Y302F plus Gαq were compared by repeated measures ANOVA and Dunnett's post-test compared to WT plus Gαq in GraphPad Prism.

### Bioluminescence Resonance Energy Transfer (BRET)

48 h after transfection, HEK293 cells were washed with PBS, detached with PBS/Trypsin-EDTA (0.25% Trypsin; 1 mM EDTA, Invitrogen), harvested by centrifugation (5 mins, 1,000g), resuspended in PBS supplemented with 0.5 mM Ca^2+^ and 0.5 mM Mg^2+^ and incubated at room temperature on a shaker (app. 250 rpm) until the time of the experiments. The resuspended cells were distributed in 96-well microplates (black/white optiplate, PerkinElmer) and incubated in the presence or absence of ligands. The reading time was 15 mins after agonist addition for dose-response curves.

DeepBlueC coelenterazine (Coelenterazine 400a, Biosynth) was added two seconds before reading using an injector at a final concentration of 5 µM. Measurement of Luc-mediated luminescence and GFP^2^-mediated emission from each well were performed using a Tecan Infinite F500 microplate reader (Tecan Group Ltd., Männedorf, Switzerland). The BRET2 ratio was determined by calculating the ratio of the light emitted by GFP^2^ (515 nm) over the light emitted by the Renilla Luc (410 nm). For BRET1, the ratio was determined by calculating the ratio of light emitted by YFP (530 nm) over the light emitted by the Renilla Luc (470 nm). The background signal from Luc was determined by co-expressing the Luc construct with empty vector, and the BRET1/BRET2 ratio generated from this transfection was subtracted from all other BRET1/BRET2 ratios. Data were analyzed in GraphPad Prism and Excel. Statistical analysis was performed in Excel using Student's t-test, paired (BRET1 studies) or unpaired (BRET2 studies), two-tailed.

## Results

To obtain new knowledge on the molecular mechanisms underlying differential signaling, we characterized rat AT1aR receptor mutants: D74N (2.50), DRY/AAY (3.49–3.51), Y292F (7.43), N298A (7.49), and Y302F (7.53) in comparison to WT, either as full length or truncated after serine 331 (331Δ), see figure 2. FLAG-tagged constructs were transiently transfected in COS-7 cells for determination of cell surface expression levels, ligand binding properties and Gα_q/11_ dependent signaling by IP accumulation. To study β-arrestin2 recruitment by BRET2, FLAG-tagged constructs were fused with Renilla Luc and transiently expressed in HEK293 cells along with GFP^2^-tagged β-arrestin2. In addition, the effect of overexpression of β-arrestin2 and Gαq protein on β-arrestin2 recruitment and G protein-dependent signaling was evaluated in transiently transfected HEK293 cells.

### Cell surface expression

**Figure 3:**
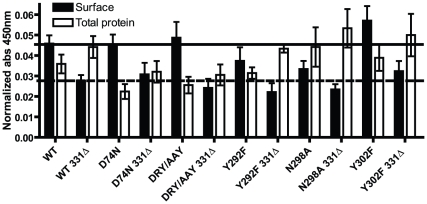
Cell surface expression determined by ELISA. Cell surface expression (black bars) and total protein levels (white bars) were determined by whole cell ELISA using M1 anti-FLAG antibody on COS-7 cells transiently transfected with FLAG-tagged rAT1aRs. Total protein levels were estimated by permeabilizing cells with 0.1% Triton-X100. Data were normalized by first subtracting values for no primary antibody and secondly subtracting values for mock transfected cells. Data are means and S.E.M.s from at least 5 independent experiments.

Cell surface expression levels were determined by whole cell ELISA using an antibody directed against the N-terminal FLAG-tag of the receptor. Results are reported in figure 3. For the full length receptors, D74N and DRY/AAY are expressed at similar levels as WT (mean ± S.E.M.: 0.046±0.004, n = 10) while Y292F and N298A show reduced expression (0.037±0.007 and 0.033±0.004, respectively, both n = 5). The Y302F mutant has a tendency to slightly higher surface expression than WT (0.057±0.007, n = 5). Truncated constructs generally display lower expression; about half of full length WT. This was similar to what previously has been found for truncated AT1Rs [28], [37]. Again, the truncated Y292F and N298A constructs have slightly lower values compared to WT 331Δ while the levels of Y302F 331Δ are slightly higher than WT 331Δ. The total protein levels vary among receptor mutants. However, the general trend is that truncated receptors show higher levels of total protein than the full length receptors. Based on competition binding studies, we have calculated the Bmax for the WT AT1R to be 24±5 fmol/10^5^ cells (mean and S.D., n = 3).

### Ang II affinity

**Figure 4:**
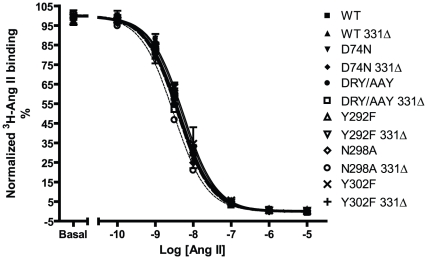
Ang II whole cell competition binding. Normalized competition binding curves from experiments using 1nM ^3^H-Ang II as tracer and increasing amounts of unlabeled Ang II. Experiments were conducted on COS-7 cells transiently transfected with FLAG-tagged rAT1aRs. WT full length and truncated receptor curves are shown in bold, curves of truncated receptors are shown as dashed lines. For each receptor, curves were normalized in GraphPad Prism by defining the fitted bottom value as 0% and top value as 100%. Normalized curves were summarized from at least 3 independent experiments performed in triplicates, means and S.E.M.s are shown. n's are reported in table 1. Experimental details can be found in the Materials and Methods section.

Affinity for the endogenous agonist Ang II was determined by whole cell competition binding using tracer amounts of ^3^H-Ang II and increasing amounts of unlabeled Ang II. Normalized curves are depicted in figure 4 and pK_D_ values are reported in table 1. The WT receptor has a pK_D_ of 8.5±0.2 (mean and S.D., n = 4). As illustrated, all of the mutants show similar affinities for Ang II compared to WT in this assay, and truncation does not appear to affect the affinity.

**Table 1:** Ligand binding.

	*pK_D_ ± S.D.*	*N*
**WT**	8.5±0.2	4
**D74N**	8.4±0.04	3
**DRY/AAY**	8.4±0.07	3
**Y292F**	8.4±0.01	3
**N298A**	8.6±0.03	3
**Y302F**	8.4±0.05	3
***WT 331Δ***	*8.5*±*0.03*	*3*
***D74N 331Δ***	*8.5*±*0.06*	*3*
***DRY/AAY 331Δ***	*8.5*±*0.2*	*3*
***Y292F 331Δ***	*8.5*±*0.06*	*3*
***N298A 331Δ***	*8.7*±*0.05*	*3*
***Y302F 331Δ***	*8.5*±*0.08*	*3*

Ang II affinity: pK_D_ values, standard deviations and n's from whole cell competition binding assay using 1nM ^3^H-Ang II as tracer and increasing amounts of unlabeled Ang II. Experiments were conducted on COS-7 cells transiently transfected with FLAG-tagged rAT1aRs in triplicate setups. Experimental details can be found in the Material and Methods section. Statistical significance was probed by one-way ANOVA and subsequent Dunnett's post-test against full length WT receptor values and no significant differences (p<0.05) were found.

### Ang II induced G protein-dependent signaling

**Figure 5:**
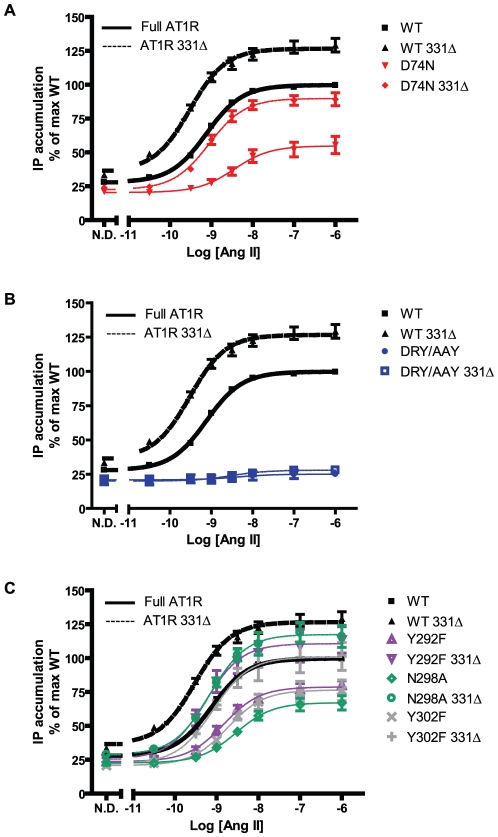
Ang II induced IP accumulation. **A**, **B, and C:** Dose-response curves normalized to the fitted maximum for WT AT1R compiled from at least 5 independent experiments performed in triplicates (mean and S.E.M.). WT and WT 331Δ have been included on all graphs for comparison. **A:** D74N. **B:** DRY/AAY. **C:** Receptor mutants: Y292F, N298A, and Y302F. Experiments were conducted on COS-7 cells transiently transfected with FLAG-tagged rAT1aR. WT full length and truncated receptor curves are shown in bold, curves of truncated receptors are shown as dotted lines. Experimental details can be found in the Material and Methods section. n's are reported in table 2. N.D. = no drug.

Ang II dose-response curves for IP accumulation of WT and mutant receptor constructs were determined. Summarized normalized curves are depicted in figure 5A–C, while pEC50 values are reported in table 2. As shown in figure 5B, the DRY/AAY is the only mutant, which appears practically abrogated in signaling capacity. pEC50 values for full length mutant receptors (except DRY/AAY, which was not possible to analyze) are significantly lower than WT (EC50 ratio 0.2–0.5, n>5, p<0.05). This supports previous reports of impaired signaling for these receptor mutants [12], [16], [17], [18], [19], [20], [30], [31]. Mutant 331Δ receptors show increased pEC50 values (∼2–4 fold change from full length) hereby showing potency similar to full length WT. None of them, however, have improvements comparable to WT 331Δ, which has a significantly increased potency (EC50 ratio 2.6, p<0.05 compared to WT). When comparing the fitted maximum values, only D74N and N298A are significantly reduced compared to WT (DRY/AAY mutants were not included in this test due to their lack of response). The DRY/AAY mutant shows abrogated signaling through the Gα_q/11_ dependent pathway (figures 5B). Signaling response - as determined by amplitude and fold over N.D. - is slightly, yet significantly increased upon truncation. However, we found no significant difference in maximum values for full length vs. 331Δ receptor mutants, and the response of the DRY/AAY 331Δ is still much reduced compared to WT. This indicates that this mutant is more G protein “uncoupled” than the other mutants.

**Table 2:** Ang II induced IP accumulation.

Receptor	pEC50 ± S.D.	Fold change compared to WT	N
**WT**	9.1±0.2	-	10
**D74N**	8.5±0.1*	0.2	5
**DRY/AAY**	N.A.	N.A.	5
**Y292F**	8.8±0.1*	0.5	5
**N298A**	8.6±0.1*	0.3	5
**Y302F**	8.8±0.2*	0.5	5
***WT 331Δ***	*9.5±0.1* *	*2.6*	*9*
***D74N 331Δ***	*9.1±0.1*	*0.9*	*5*
***DRY/AAY 331Δ***	*N.A.*	*N.A.*	*5*
***Y292F 331Δ***	*9.3±0.1*	*1.4*	*5*
***N298A 331Δ***	*9.2±0.1*	*1.2*	*5*
***Y302F 331Δ***	*9.1±0.2*	*1*	*5*

pEC50 values and standard deviations from dose-response curves of induced IP accumulation on COS-7 cells transiently transfected with FLAG-tagged rAT1aRs stimulated with Ang II for 45 mins. Fold change values of EC50 are reported for comparison between WT and mutant receptors. Data are summarized from at least 5 independent experiments performed in triplicates. Experimental details can be found in the Material and Methods section.

*indicates p<0.05 determined by one-way ANOVA and subsequent Dunnett's post-test against full length WT receptor values.

In summary, mutant receptors are all impaired in the IP accumulation assay, however, not to the same degree. Truncation increases potency and efficacy indicating that desensitization is involved in the impaired response, but is not completely able to rescue the phenotype of these mutants. Also, only the DRY/AAY mutant appears severely impaired in signaling in our setup.

### β-arrestin2 recruitment in response to Ang II

**Figure 6:**
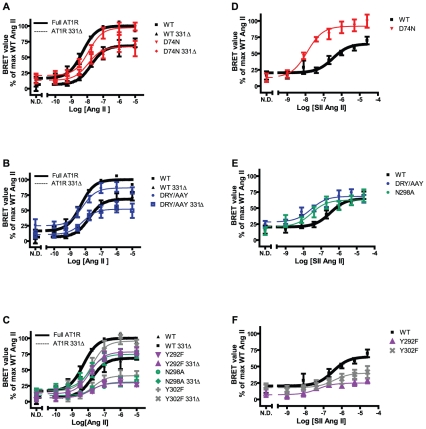
β-arrestin2 recruitment by BRET. Dose-response curves normalized to the fitted maximum for WT AT1R stimulated with Ang II compiled from at least 4 independent experiments performed in duplicate (mean and S.E.M.). All values are shown as percent of the maximal response with Ang II on WT AT1R. WT AT1R and WT 331Δ AT1R have been included on graphs for comparison. **A–C:** Stimulation with AngII. **C**, **D–F:** Stimulation with SII Ang II. **A:** D74N. **B:** DRY/AAY. **C:** Y292F, N298A, and Y302F. **D:** D74N. **E:** Receptor mutants: DRY/AAY and N298A. **F:** Receptor mutants: Y292F and Y302F. Experiments were conducted on HEK293 cells transiently co-transfected with Luc-tagged rAT1aRs in combination with GFP2-tagged β-arrestin2. Curves of full length receptors are shown as full lines, and curves of truncated receptors are shown as dashed lines. WT full length and truncated receptor curves are shown in bold. N.D. = no drug. Experimental details can be found in the Material and Methods section. n's are reported in table 3.

To study the ability of mutant receptors to recruit β-arrestin2, we co-transfected C-terminally Luc-tagged AT1R constructs with N-terminally GFP^2^-tagged β-arrestin2 and studied ligand-induced association between the molecules using a BRET assay. Compiled curves are depicted in figures 6A–C and pEC50 and maximum response values in table 3. For the WT receptor, Ang II induces a robust dose-dependent recruitment, which is impaired in potency and efficacy for the truncated WT construct (ratios 0.3 and 0.8 compared to full length, respectively). Somewhat unexpectedly, only the DRY/AAY mutant has similar potency as WT while the others are impaired (ratios 0.2–0.4) compared to WT (p<0.05). Truncation only affects Ang II potency for the D74N mutant – which actually is more potent than the full length receptor. Truncation, however, significantly impairs maximal responses of the receptor mutants (ratios 0.6-0.8). All of the full length receptor mutants except N298A show maximum responses similar to WT in the β-arrestin2 recruitment assay, see figure 6A–C and table 3.

**Table 3:** β-arrestin2 recruitment measured by BRET.

*β-arrestin2 recruitment*	*Ang II*				*SII Ang II*			
	*pEC50*	*Fold change*	*Maximum Response*	*n*	*pEC50*	*Fold change*	*Maximum response*	*n*
WT	8.4±0.2	-	0.20±0.03	5	6.5±0.2	-	0.15±0.02	4
D74N	7.9±0.1*	0.4	0.20±0.02	5	7.8±0.3*	23	0.19±0.02*	5
DRY/AAY	8.2±0.1	0.7	0.18±0.02	5	7.5±0.1*	11	0.15±0.01	4
Y292F	7.9±0.3*	0.4	0.17±0.01	5	N.R.	-	N.R.	5
N298A	7.7±0.5*	0.2	0.17±0.01*	5	7.9±0.2*	23	0.15±0.02	4
Y302F	7.7±0.2*	0.2	0.19±0.01	5	N.R.	-	N.R.	5
*WT 331Δ*	*7.8*±*0.2* *	*0.3*	*0.16*±*0.01* *	*4*	*6.6*±*0.3*	*1.3*	*0.12*±*0.02* *	*5*
*D74N 331Δ*	*8.1*±*0.1* §	*0.6*	*0.14*±*0.02* * §	*4*	*7.5*±*0.2* *	*11*	*0.15*±*0.02* §	*5*
*DRY/AAY 331Δ*	*8.2*±*0.3*	*0.7*	*0.15*±*0.03* * §	*4*	*7.0*±*0.3* †	*3.6* †	*0.15*±*0.03*	*4*
*Y292F 331Δ*	*7.7*±*0.2* *	*0.2*	*0.11*±*0.01* * §	*4*	*N.R.*	*-*	*N.R.*	*5*
*N298A 331Δ*	*7.6*±*0.1* *	*0.2*	*0.12*±*0.01* * §	*4*	*7.1*±*0.3* †	*4.3* †	*0.12*±*0.01*	*4*
*Y302F 331Δ*	*7.6*±*0.2* *	*0.2*	*0.12*±*0.01* * §	*4*	*N.R.*	*-*	*N.R.*	*5*

pEC50 values and standard deviations from dose response curves of ligand induced β-arrestin2 recruitment on HEK293 cells transiently co-transfected with Luc-tagged rAT1aRs and GFP^2^-tagged β-arrestin2 stimulated with Ang II or SII Ang II for 15 minutes. Fold change EC50 values compared to full length WT receptor are reported. Maximum response and standard deviations reported from dose response curves. Data are summarized from at least 4 independent experiments performed in duplicates. Experimental details can be found in the Material and Methods section.

*indicates p<0.05 determined by two-tailed Student's t-test against full length WT receptor values.

§ indicates p<0.05 determined by two-tailed Student's t-test against the full length version of WT/mutant receptor.

† pEC50 values for DRY/AAY 331Δ and N298A 331Δ with SII Ang II are determined from normalized dose-response curves. Fold responses of these two receptor mutants are determined from the WT pEC50 values for normalized curves of SII Ang II: 6.5±0.3 for full length and 5.8±0.3 for 331Δ.

### SII Ang II induced β-arrestin2 recruitment

Previous studies of G protein-independent β-arrestin recruitment using SII Ang II has shown that this recruitment is weaker than for Ang II (∼60%), most likely because G protein activation increases the ability of the receptor to recruit β-arrestins [34], [38]. Since all of our mutants are impaired in G protein-dependent signaling, this might be the reason why the potency of Ang II induced recruitment of β-arrestin is impaired compared to WT. We therefore measured dose-response curves of SII Ang II induced β-arrestin2 recruitment (table 3 and figures 6D–F). This characterization reveals three very interesting phenotypes for the mutants. *The D74N mutant* has significantly increased potency (∼23 fold) and maximum response (∼1.3 fold) compared to WT (figure 6D). *The DRY/AAY and N298A mutants* show significantly increased potencies for SII Ang, but maximum responses similar to WT (figure 6E). *Receptor mutants Y292F and Y302F* are substantially impaired in recruitment of β-arrestin2 in response to SII Ang II (figure 6F). The truncated mutants generally follow the tendencies of their full length equivalents (data not shown).

### SII Ang II induced IP accumulation

**Figure 7:**
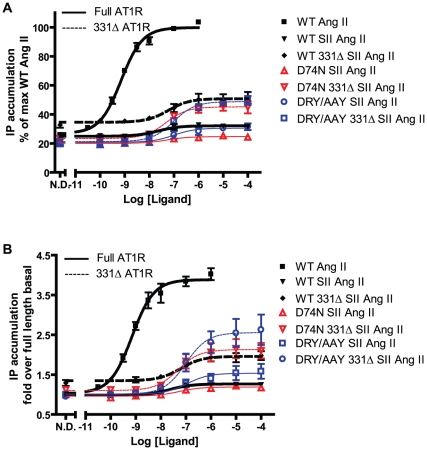
SII Ang II induced IP accumulation. **A:** Dose-response curves normalized to the fitted maximum response for WT AT1R compiled from at least 3 independent experiments performed in triplicates (mean and S.E.M.). **B:** Dose-response curves normalized to no drug (N.D.) response for the full length receptor. In this graph, one data set for DRY/AAY 331Δ had to be left out due to missing values for full length DRY/AAY on that particular assay date. Experimental setup as in figure 5.

To ensure that the increased signaling capabilities of the mutants compared to WT receptor was specific for β-arrestin2 recruitment, we tested dose-response properties of the receptor mutants D74N, DRY/AAY, N298A in the IP assay (figure 7 (WT, D74N and DRY/AAY) and Figure S1 (N298A)). As previously reported, SII Ang II does not induce signaling from WT [21], nor do the D74N and N298A mutants respond to this ligand. Truncation increases the signaling response for all constructs tested. Interestingly, SII Ang II appears to be slightly more efficacious on the DRY/AAY mutant full length and truncated than on the WT and D74N, when looking at the response over “baseline”, figure 7B. The maximum response is, however, still much lower than that of WT in response to Ang II.

### SII Ang II affinity

**Figure 8:**
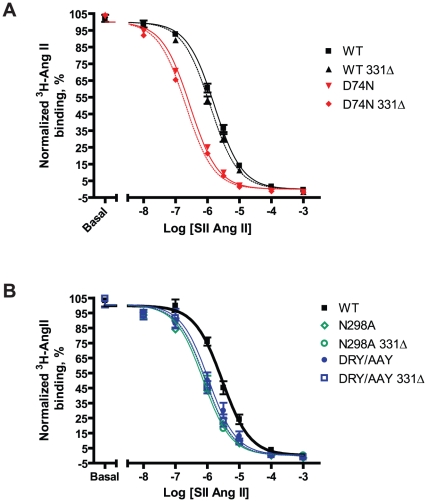
SII Ang II affinity. Normalized competition binding curves from experiments using 1nM ^3^H-Ang II as tracer and increasing amounts of unlabeled SII Ang II. Experiments were conducted on COS-7 cells transiently transfected with FLAG-tagged rAT1aRs. WT full length and truncated receptor curves are shown in bold, curves of truncated receptors are shown as dashed lines. For each receptor, curves were normalized in GraphPad Prism by defining the fitted bottom value as 0% and top value as 100%. Normalized curves were summarized from at least 3 independent experiments performed in triplicates, means and S.E.M.s are shown. pKi values and n's are reported in table 4. Experimental details can be found in the Material and Methods section. **A:** WT and D74N. **B:** DRY/AAY and N298A.

To investigate whether the increased potency of SII Ang II for the mutants D74N, DRY/AAY and N298A was caused by increased affinity for SII Ang II, we studied SII Ang II affinity by heterologous competition binding using tracer amounts of ^3^H-Ang II and unlabeled SII Ang II. Results are reported in figure 8 and table 4. We found that all three mutants show significantly increased affinity for SII Ang II compared to the WT in the range of 3–5.5 fold.

**Table 4:** Whole cell competition binding – SII Ang II.

*SII Ang II*	pK_i_±S.D.	*n*	*Fold change to WT*
**WT**	5.9±0.09	4	-
**D74N**	6.6±0.06*	4	5.5
**DRY/AAY**	6.1±0.2*	3	3.0
**N298A**	6.2±0.04*	3	4.1
***WT 331Δ***	*6.0*±*0.08* *	*4*	*1.4*
***D74N 331Δ***	*6.8*±*0.06* *	*4*	*7.8*
***DRY/AAY 331Δ***	*6.2*±*0.2* *	*3*	*4.0*
***N298A 331Δ***	*6.2*±*0.06* *	*3*	*4.0*

pK_i_ values have been calculated based on K_D_ values for homologous competition with Ang II executed in parallel with the SII Ang II experiments. DRY/AAY and N298A mutants (full and truncated) have been tested in a series of experiments separate from D74N mutants. WT pKi (full and 331Δ) are reported for D74N series in the table. For the experiments with DRY/AAY and N298A mutants WT pK_i_ was: 5.6±0.1 (mean and S.D., n = 3). Statistics were carried out separately for each set of experiments. Both datasets were analyzed by repeated measures ANOVA with subsequent Dunnet's multiple comparisons test against WT pK_i_ in GraphPad Prism.

*indicates P<0.05.

### Effect of β-arrestin and G protein overexpression on IP accumulation and β-arrestin2 recruitment

To further test our hypothesis that a biased signaling phenotype can result from competition between signaling pathways rather than direct inability to interact with a given downstream signaling molecule, we tested the effect of G protein overexpression in HEK293 cells transiently transfected with receptors and β-arrestin2 in amounts typically used to measure β-arrestin2-recruitment with BRET1.

**Figure 9:**
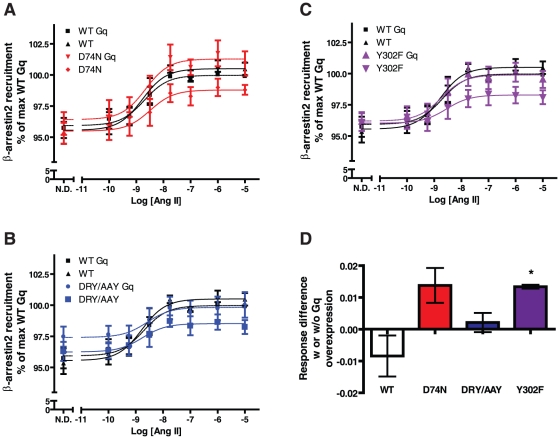
β-arrestin2 recruitment with G protein overexpression. Dose-response curves of Ang II-induced β-arrestin2 recruitment determined by BRET1. Normalized curves compiled from four independent experiments performed in triplicates and standard errors are shown. Differences in response between cells overexpressing Gαq protein or not were measured by BRET1. Response is defined as: no drug value subtracted from average of 10^−6^ and 10^−5^ M Ang II values. * indicates p<0.05 determined by paired Student's t-test to WT values.

Data on β-arrestin2-recruitment in response to Ang II are reported in figure 9. The WT receptor shows similar or slightly lower response with overexpression of Gαq. The D74N mutant shows a tendency towards increase in response upon Gαq overexpression compared to WT, while the difference response for the Y302F mutant is significantly greater than for the WT (0.013±0.0006 vs. -0.008±0.006, mean and S.E.M., n = 4). In contrast, the difference in response upon G protein overexpression is not very large for the DRY/AAY mutant – again supporting that this mutant has a conformation different from the other receptor mutants.

**Figure 10:**
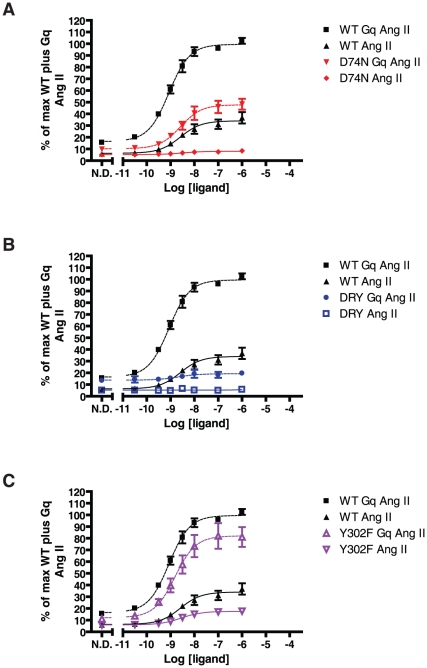
IP accumulation with overexpression of β-arrestin2 and G protein. Dose-response curves of Ang II stimulation normalized to the fitted maximum for WT AT1R plus G protein compiled from four independent experiments performed in triplicates (mean and S.E.M.). WT has been included on all graphs for comparison. **A:** D74N. **B:** DRY/AAY. **C:** Y302F. Experiments were conducted on transiently transfected HEK293 cells. N.D. = no drug.

When looking at G protein-dependent signaling depicted in figure 10, signaling is drastically impaired by the overexpression of β-arrestin2. Overexpression of Gαq protein increases Ang II-induced signaling for all of the tested receptors except the DRY/AAY mutant, which remains practically uncoupled. In the cells overexpressing G protein, the D74N mutant still has a pEC50 value significantly lower than the WT (D74N 8.6±0.1 vs. WT 9.0±0.2; mean and S.D., n = 4), while the Y302F is not significantly different though there is a trend towards it being lower than the WT (8.8±0.1, n = 4). Similarly, there is a tendency towards increased pEC50 for the WT receptor upon G protein overexpression (8.7±0.3), which is also not statistically significant. Hereby, these results are similar to the IP accumulation data for full length and truncated constructs and further support that different mechanisms of G protein uncoupling exist. For SII Ang II stimulation, signaling is slightly increased (basal levels and responsiveness) for WT, DRY/AAY and Y302F mutants upon overexpression of G protein, see figure S2. However, efficacy remains practically nonexistent.

## Discussion

**Figure 11:**
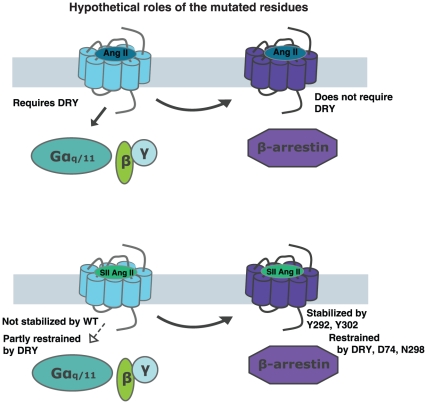
Schematic overview of different phenotypes of the mutants. Schematic presentation of how the residues mutated in this study could hypothetically affect the induction of different conformations. These interpretations are based on the data included in this study and should be considered with the precautions mentioned in the Discussion section.

In this study, we investigated the mechanistic background of five AT1R receptor mutants previously published to be biased towards G protein-independent pathways. We characterized their signaling through Gα_q/11_ versus β-arrestin2 in response to Ang II and SII Ang II and probed of the effect of their stable interaction with β-arrestins on their signaling phenotypes. Also, we tested the effect of overexpression of intracellular signaling partners for select mutants. The results strongly support our hypothesis that the receptor mutants are uncoupled from G protein-dependent signaling by principally different mechanisms, which are most likely caused by distinct conformational states. We have sought to summarize the hypothetical conformations induced by the different mutations in figure 11.

Based on our results, the mutants can be categorized into four different groups discussed below. *First*, *the DRY/AAY mutant* is severely impaired in Ang II induced G protein-dependent signaling, which is not reversed upon truncation or overexpression of G protein. This finding is in good correspondence with other studies of the AT1R [12], [17], [39], [40], [41], [42]. The crystal structure of opsin bound to a peptide derived from the C-terminal of transducin suggests that R3.50 (of the DRY motif) is important for the interaction with G protein while the D3.49 is important for the transition between inactive and active states [43]. In contrast, the receptor mutant shows robust increased potency for SII Ang II in the β-arrestin2 recruitment assay, which is likely explained by its increased affinity for SII Ang II, but could also suggest a bias towards β-arrestin-dependent signaling, as discussed below. Surprisingly the mutant appeared more prone to SII Ang II activated signaling through Gα_q/11_ than WT suggesting that the conformations induced by SII Ang II and by the DRY/AAY mutation are not the same and can partly complement each other. Hereby, it also demonstrates that the DRY motif is not absolutely necessary for AT1R mediated activation of G proteins. However, the level of G protein-dependent activation was still very low and we did not observe this in the overexpression experiments. Thus, further studies will be needed to clarify the exact mechanisms involved.

*Second, the D74N mutant* shows impaired G protein-dependent signaling, which is restored to full length WT levels upon truncation, and shows >20 fold increased potency and increased efficacy for β-arrestin2 recruitment in response to SII AngII. This is not accompanied by a change in potency and efficacy for SII Ang II signaling through Gα_q/11_. We propose that at least part of the mechanism underlying this phenotype is a relative preference for β-arrestin2 recruitment rather than inability to interact with G protein. The mutant shows increased affinity for SII Ang II, which is in agreement with previous studies [16]. However, we do not believe that the increased binding affinity for SII Ang II can fully explain the changes in potency/efficacy profiles for SII Ang II in the BRET vs. IP accumulation assay. Indeed, the increased affinity for peptide analogues and the decreased affinity for inverse agonists previously reported for D74 mutants may reflect that the mutant shows constitutive association with β-arrestin2, which has been reported to form a high affinity ternary complex, similar to the G protein bound receptor state [44], [45]. Further studies will be needed to confirm this.

*Third, the N298A mutant* is moderately impaired in signaling through Gα_q/11_ showing WT-like efficacy when normalized to surface expression. The receptor mutant shows increased potency for SII Ang II induced β-arrestin2 recruitment and increased SII Ang II affinity similar to what was found for the DRY/AAY mutant. This mutant does not show any Gα_q/11_ induced signaling in response to SII Ang II. *Fourth, Y292F and Y302F mutants* display moderately impaired G protein-dependent signaling, and severely impaired recruitment of β-arrestin2 in response to SII Ang II. This could suggest that SII Ang II induces a conformation in these mutations that is different from that of the WT and the two other mutations. Murine AT1aR Y292F has previously been found unable to induce ERK1/2 phosphorylation in response to SII Ang II [46], which is in good agreement with our findings. The authors suggest that Y292 (7.43) is required for SII Ang II to induce the conformation able to activate ERK1/2, which appears to be extendable to β-arrestin2 recruitment. Our studies suggest that the Y302F mutation may have a similar function.

Previous studies have shown severely compromised G protein-dependent signaling of some of the mutant receptors [16], [18], [30], [31], while we find most of them to be only moderately impaired. This discrepancy is most likely due to differences in the method for detection of IP accumulation such as time of stimulation, which is shorter in the aforementioned studies. It is likely that this also reflect impaired kinetics of these receptor mutants compared to WT [17]. Cellular levels of receptors and signaling components such as β-arrestins may also contribute to the discrepancy. Our results do, however, allow us to separate the phenotypes of the receptor mutants yielding only the DRY/AAY mutant as abrogated in Ang II induced G protein-dependent signaling and largely unaffected by truncation as well as overexpression of G protein.

The BRET assay offers the possibility of studying protein-protein interactions in response to ligands in live cells. However, there are certain caveats to the assay that have to be considered. It is not possible to distinguish between signal from receptors being functionally expressed on the surface and receptors trapped in the ER during biosynthesis, which can be a problem for receptor mutants. This can lead to a false decrease in BRET ratios affecting both potency and efficacy. However, this does not necessarily affect the observed potency changes observed for Ang II and SII Ang II for the different mutations. Maximum responses in the BRET assay must also be interpreted carefully since this response is very sensitive to conformational changes of the receptor-Luc molecule, which could be introduced by the truncation and mutation. Yet, the importance of the C-terminal tail of the AT1R in β-arrestin recruitment has been demonstrated several times in the literature using other methods to detect the receptor-β-arrestin interactions [27], [29], [47].

Differential activation of signaling pathways can also be mediated at the cellular level by the availability of implicated signaling proteins and the kinetics of activation of the different signaling pathways [48]. Thus, these factors are important to take into account when interpreting data on biased signaling. In our studies, we have studied G protein-dependent activation of the inositol phosphate signaling pathway. IP accumulation provides a good measure of G protein activation. However, there may be certain pitfalls in measuring a downstream signaling pathway instead of direct receptor-G protein interaction, and such an analysis could provide additional insights to the mechanisms involved. On a similar note, characterization of G protein-independent signaling pathways, e.g. ERK1/2 activation, would also provide interesting insights to the different phenotypes of the receptor mutants. Though it is well-established that recruitment of β-arrestin2 facilitates ERK1/2 activation for the AT1R, β-arrestin2 recruitment does not necessarily reflect activation of β-arrestin-arrestin dependent signaling [7]. Such studies are, however, easily complicated since by the complex nature of G protein-independent signaling, which remains to be fully mapped [49], [50], just as both dose and stimulation time required to yield the maximal response could be affected by mutation.

Though it is now generally recognized that multiple signaling conformations exist and may be physiologically relevant for a number of 7TMRs [1], [6], the molecular determinants underlying specific G protein or β-arrestin interactions remain largely unresolved.

With the exception of the Y292 (7.43) residue, the residues included in this study are all highly conserved in Family A 7TMRs [51]. The residues are proposed to form polar interactions involving water molecules that are important for receptor activation, including interaction between positions 2.50 (D74) and 7.49 (N298A) based on evidence from crystal structures, but also a number of mutational studies including one of the AT1R [52], [53], [54], [55], [56], [57], [58]. Except for the DRY motif, which is now fairly recognized as being specifically important for G protein-coupling, the data on G protein-independent pathways or desensitization for the remaining mutants is scarce for other 7TMRs. Mutation of residues in the NPXXY motif were found to impair signaling as well as internalization in the β2-adrenergic receptor [59]. Correspondingly, in the Formyl Peptide Receptor mutations of D2.50 and N7.49 inhibited internalization and β-arrestin recruitment [60]. Here, we found different phenotypes for the N7.49 and the Y7.53 receptor mutants, N7.49 showing increased potency of SII Ang II induced β-arrestin2 recruitment and Y7.53 being impaired for this function. The existing crystal structures suggest an important function of Y7.53 as a rotameric switch that participates in an aromatic interaction with helix 8 in inactive rhodopsin [3], [4], [61]. This amino acid is part of an interhelical hydrogen-bonding network in the “non-rhodopsin” structures, and is involved in keeping TM6 in its outward position in the structures of opsin. Further studies will be needed to the exact roles of the TM2 and TM7 residues in induction of differentially activating phenotypes.

The concept of differential signaling or biased agonism challenges the traditional understanding of how 7TMRs exert their physiological roles and offers the possibility to develop novel treatment options selectively targeting specific signaling pathways. To do so, it is, however, crucial to understand the molecular mechanisms behind the phenomenon. Our study proposes that selective G protein-uncoupling leading to selective β-arrestin-dependent signaling can occur both through specific conformations lacking epitopes necessary for G protein-coupling and through conformations with a “gain of function” for β-arrestin recruitment compared to WT. Since induction of such different conformations by external ligands in an *in vivo* setting might not lead to similar outcomes, it will be of utmost importance to be able to distinguish between such conformations at the molecular level. Another interesting perspective to this study is that lack of epitopes important for G protein-coupling can be at least partially compensated in certain settings -exemplified in our study by the effects of SII Ang II on the DRY/AAY mutant. This latter finding is in good agreement with the current theory of 7TMR activation being mediated by different micro-switches working together in an allosteric fashion [3]. Further studies of the molecular mechanisms underlying biased agonism and development of more biased agonists will be needed to address these issues.

In conclusion, even seemingly similar phenotypes in differential signaling may represent principally different mechanisms of G protein-uncoupling, which again are most likely caused by distinct conformational states. We show how a strong interaction with β-arrestin2 might affect G protein-dependent signaling responses. Hereby, the results obtained in this study have implications for the design and interpretation of future studies of the molecular determinants of differential signaling of 7TMRs.

## Supporting Information

Figure S1 SII Ang II induced IP accumulation. A: Dose-response curves of IP accumulation from N298A mutants normalized to the fitted WT Ang II induced maximum response. B: Curves normalized to full length no drug (N.D.) response. WT Ang II has been included as a control in bold. Dashed line indicates truncated receptor. Experimental setup as in figure 5.
(1.13 MB EPS)

Figure S2 IP accumulation with overexpression of β-arrestin2 and G protein. Dose-response curves of SII Ang II stimulation normalized to fitted maximum WT plus G protein Ang II compiled from three independent experiments performed in triplicates. WT curves have been included on all graphs for comparison. Dashed lines indicate overexpression of Gαq protein. Experiments were performed on transiently transfected HEK293 cells.
(1.41 MB EPS)
